# Special Issue “25 Anniversary of Bioinformatics of Genome Regulation and Structure Conference Series”

**DOI:** 10.3390/ijms26136447

**Published:** 2025-07-04

**Authors:** Yuriy L. Orlov, Nina G. Orlova, Ming Chen, Nikolay A. Kolchanov

**Affiliations:** 1 The Digital Health Center, I.M. Sechenov First Moscow State Medical University of the Ministry of Health of the Russian Federation (Sechenov University), 119991 Moscow, Russia; 2 Institute of Cytology and Genetics, Siberian Branch of the Russian Academy of Sciences, 630090 Novosibirsk, Russia; 3 Novosibirsk State University, 630090 Novosibirsk, Russia; 4 Department of Mathematics, Financial University Under the Government of the Russian Federation, 125167 Moscow, Russia; 5 Department of Bioinformatics, College of Life Sciences, Zhejiang University, Hangzhou 310058, China

This article overviews historical trends in studies on gene expression regulation published in “25 Anniversary of Bioinformatics of Genome Regulation and Structure Conference Series” (https://www.mdpi.com/journal/ijms/special_issues/0LGA6103S5, accessed on 1 July 2025) Special Issue based on experience of the BGRS (Bioinformatics of Genome Regulation and Structure) conference series. The 25th anniversary of the BGRS conferences passed in 2023 when this Special Issue was organized, counting from the first meeting in Novosibirsk, Russia, in 1998. BGRS is the longest-running biannual international conference series gathering scientists, bioinformaticians, medical doctors, and geneticists in Novosibirsk every two years (see the archive of the conference materials at https://bgrssb.icgbio.ru/2024, accessed on 1 July 2025). The key scientific topic to be considered is the regulation of gene expression at the transcriptional level. This topic, together with BGRS conference discussions, has been presented at the journal paper collections at *Frontiers in Genetics* (https://www.frontiersin.org/research-topics/40408/bioinformatics-of-genome-regulation-and-systems-biology-volume-iii, https://www.frontiersin.org/research-topics/21036/high-throughput-sequencing-based-investigation-of-chronic-disease-markers-and-mechanisms, accessed on 1 July 2025), as well as in the publication series at *BioMedCentral* journals (https://bmcgenomics.biomedcentral.com/articles/supplements/volume-20-supplement-3, accessed on 1 July 2025) [1,2]. *IJMS* has already published several successful Special Issues on the topic related to the BGRS conference series, including “Molecular Mechanisms of Gene Expression: “Bioinformatics of Gene Regulations and Structure””, “Bioinformatics of Gene Regulations and Structure–2022” (https://www.mdpi.com/journal/ijms/special_issues/Bioinformatics_Gene, accessed on 1 July 2025), and earlier Special Issues [3]. Recently we overviewed the current state of art in these research areas [1,3].

Here we note the fundamental studies presenting research development of the bioinformatics school in the past years and new studies highlighting recent achievements in the gene expression regulation area. This Special Issue collected the articles on bioinformatics from the Institute of Cytology and Genetics of the Siberian Branch of the Russian Academy of Sciences (ICG SB RAS) in Novosibirsk, Russia, presented at the BGRS\SB-2024 anniversary meeting. Discussing bioinformatics, we commemorate other important international symposia on genetics organized by the ICG SB RAS, such as the memorial Belyaev conference on genetics and evolution «Belyaev Readings–2017» (http://conf.bionet.nsc.ru/belyaev100/en, accessed on 1 July 2025). The full member of the USSR Academy of Sciences, Professor Dmitry K. Belyaev (1917–1985), was the founder and first director of the Institute [4]. D.K. Belyaev was an outstanding scientist, evolutionist, and geneticist who investigated the background on the genetics of behavior and the domestication of wild animals [5,6,7]. We note the works that laid the basics of gene expression research conducted by Prof. Vladimir K. Shumny on plant genetics [8,9], Prof. Vadim A. Ratner on mathematical biology of gene model [10,11,12,13], and Dr. Vitaly A. Likhoshvai on gene network theory [14,15]. Thus, the research directions of gene expression and genome regulation discussed at the BGRS series [13,14] include sequence analysis, transcription regulation, gene network modeling, and medical applications of gene function analysis [3]. In addition, this *IJMS* Special Issue continued the trend set by the earlier journal issues and papers collections of advances in computer genomics and bioinformatics presented at Young Scientist Schools “Systems biology and bioinformatics” (SBB)—education courses series [16,17].

We open this collection of papers with the work by Anton Bogomolov and co-authors (https://doi.org/10.3390/ijms25010607, accessed on 1 July 2025) on gene promoters’ analysis and nucleotide polymorphisms [contribution 1]. Gene expression regulation studies at the transcription level were the key research area for the BGRS conference series from the beginning, in 1998 [18]. This topic has been presented in a series of works on TATA-box binding mechanisms [19,20]. The latest model uses SNP (single nucleotide polymorphisms) in the TATA box from an open bioinformatics database and the tool to reveal molecular markers in plant proximal promoters [20]. Protein transcriptional factors regulate gene expression at the transcription level. Experimentally, the binding of regulatory transcription factors can be detected by ChIP-seq (chromatin immunoprecipitation followed by sequencing) technology [21]. The article by Oleg Vishnevsky and colleagues (https://doi.org/10.3390/ijms25021011) continued the topic of transcription factor binding analysis, investigating the sequences in ChIP-seq peaks [contribution 2]. The specificity of transcription factors binding in the regulatory gene regions underlies gene expression machinery. The studies on sequence specificity and interplay between transcription factor binding were studied by the authors from ICG SB RAS in the early 2000s [22] and continued in recent ChIP-seq studies [23,24].

The next block of articles in this Special Issue [contributions 3–6] presents medical applications and cancer studies. Justin Hummel et al. (https://doi.org/10.3390/ijms25063220) identified genomic signatures for colorectal cancer survival [contribution 3]. The authors used a novel exploratory algorithm [25] based on artificial intelligence (explainable artificial intelligence, XAI) that integrates mutational and clinical features to identify genomic signatures. Thus, this work confirmed the trend of AI tools and algorithms in bioinformatics as a priority area for gene expression research [3,26]. Tyler Kwok and colleagues (https://doi.org/10.3390/ijms25105439) analyzed genes crucial for biological processes in breast cancer [contribution 4]. The authors used a comprehensive approach integrating pathway analysis, overall survival analysis, identification of differentially expressed genes, visualization of the protein–protein interaction network, and transcription factor analysis. Thus, we would note the network approach for gene regulation studies [13,14]. The next article by Styliani Geronikolou and co-authors (https://doi.org/10.3390/ijms25147684) presented the reconstruction of the interactome of non-hereditary obesity, followed by a drug target search for this complex disorder [contribution 5]. The network approach led to the generation of an obesity interactions network (“obesidome”) [27]. The cholesterol biosynthesis gene network overlaps with the non-hereditary obesity network [28]. Hongquan Chen et al. (https://doi.org/10.3390/ijms25158096) studied the kidney renal clear cell carcinoma prognostic model [contribution 6]. The authors noted considerable progress in the application of artificial intelligence algorithms in the medical field, particularly in cancer diagnosis [26,29].

Another set of articles [contributions 7–9] highlights recent gene expression studies in the brain in animal models. Indeed, direct molecular studies of cognitive dysfunction on human brain tissues are not possible. Genetic models on mice and rats were actively developed at ICG SB RAS starting from founding works by Academician D.K. Belyaev [5,7]. Using a high-fat induced diabetic mouse model, Zhaoming Cao and co-authors (https://doi.org/10.3390/ijms25041990) studied N6-methyladenosine modification patterns that contribute to cognitive dysfunction [contribution 7]. The diabetic mouse model is actual for cognitive dysfunction research because patients with type 2 diabetes mellitus are more likely to develop mild cognitive impairment and associated diseases. Vladimir Babenko et al. (https://doi.org/10.3390/ijms25052882) studied major pathways related to chronic stress in animals using RNA-seq [contribution 8]. This work continues animal stress studies at ICG SB RAS in Novosibirsk based on next-gen sequencing [30]. Models of animal domestication are also related to stress. The selection of animals showing tame or aggressive behavior toward humans has become the basis of research started by D.K. Belyaev back in 1958 on farm foxes; later, it was continued on minks and gray rats [6,7]. These animal models provide unique data for modern gene expression research [31,32]. Irina Chadaeva and colleagues (https://doi.org/10.3390/ijms25094613) studied differential gene in brain regions of gray rats selected for tame or aggressive behavior [contribution 9]. The authors defined differentially expressed genes in the rat brain regions related to behavior patterns. Using the same rat model, the role of transcription factors as regulators of changes in behavior through domestication has been shown previously by this authors’ group [33]. We close the section of gene expression studies on animal models by application in fish. Martyna Arciuch-Rutkowska et al. (https://doi.org/10.3390/ijms25094619) analyzed the effect of dietary supplementation on growth performance and expression of immune-related genes in juvenile African catfish (*Clarias gariepinus*) [contribution 10]. We authors used metagenomic sequencing of the intestinal contents of the catfish.

The final block of papers [contributions 11–13] highlights plant biology bioinformatics and genome structure analysis. Regulation of the organelle gene expression is crucial for plant growth and development. Igor Gorbenko et al. (https://doi.org/10.3390/ijms25158164) studied transcriptome changes and faster growth in Arabidopsis [contribution 11]. The length of mitochondrial genomes varies significantly among plant species and can expand through the replication of its sequences and the transfer of nuclear genome fragments and chloroplast genome fragments, raising the problem of repeated sequences. Xiaofan He and colleagues (https://doi.org/10.3390/ijms242417334) analyzed structural reorganization in two alfalfa mitochondrial genome assemblies and mitochondrial evolution in *Medicago* species, integrating sequencing data [contribution 12]. Gene expression in plants interplays with metagenome state. The microbiome has crucial functions to support plant growth and development. The review paper by Ivan Petrushin and co-authors (https://doi.org/10.3390/ijms25020750) discovers the current state of the art for potato microbiome studies [contribution 13]. The incorporation of metabolomic and proteomic data in metagenomic analysis could lead to a more profound understanding of plant defense mechanisms.

We sum up the review of BGRS conference materials and current trends in bioinformatics, noting throughput sequencing and post-genome technologies as modern instruments and data sources. The BGRS conference series continues initiating science discussions on perspective research fields, such as interdisciplinary studies in computational genomics of model organisms—plants, animals, and their microbiome. The complex network interactions, the systems biology approaches, are now more oriented toward machine learning and AI (artificial intelligence) tools [34].

Thus, a variety of model species has to be studied by sequencing methods exploring the gene expression regulation topic [35]. Machine learning models and artificial intelligence applications present contemporary trends in gene expression studies [25,34]. Integration of bioinformatics data is a challenge for deeper insights into complex biological systems and data retrieval, as was discussed at the recent Integrative Bioinformatics conference [36], a parallel international event for BGRS-2024.

To extend the current overview of the gene expression and genome regulation topic, we note an open Special Issue on plant bioinformatics, titled “Plant Biology and Biotechnology: Focus on Genomics, Bioinformatics and AI” (https://www.mdpi.com/journal/ijms/special_issues/1D646O0T7V, accessed on 1 July 2025). Problems of gene expression from a biophysical point of view will be presented during the open call for the *Biophysical Reviews* journal (“Modern Biophysical Methods: Insights from the Russian Autumn School on Biophysics in Kazan 2024”) (https://link.springer.com/collections/ifbbihjicj, accessed on 1 July 2025). We continue the series of collated papers with the *Gene Expression* Special Issue (https://www.xiahepublishing.com/journal/ge/features/bioinformatics, accessed on 1 July 2025). The topic “Bioinformatics of Gene Regulations and Structure–2025” (https://www.mdpi.com/journal/ijms/special_issues/3I56FG1O3A, accessed on 1 July 2025) continues the series of collections on gene expression. To follow readers’ interest, we will continue to collect papers on bioinformatics of gene expression for ongoing topical issues.
